# Case report: Management of pediatric gigantism caused by the TADopathy, X-linked acrogigantism

**DOI:** 10.3389/fendo.2024.1345363

**Published:** 2024-02-28

**Authors:** Manuela Caruso, Diego Mazzatenta, Sofia Asioli, Giuseppe Costanza, Giampaolo Trivellin, Martin Franke, Dayana Abboud, Julien Hanson, Véronique Raverot, Patrick Pétrossians, Albert Beckers, Marco Cappa, Adrian F. Daly

**Affiliations:** ^1^ Department of Pediatrics, Azienda Policlinico Università di Catania, Catania, Italy; ^2^ Department of Biomedical and Neuromotor Sciences (DIBINEM), Alma Mater Studiorum, University of Bologna, Bologna, Italy; ^3^ Istituto di Ricovero e Cura a Carattere Scientifico Istituto delle Scienze Neurologiche di Bologna, Bologna, Italy; ^4^ Department of Biomedical Sciences, Humanitas University, Milan, Italy; ^5^ Istituto di Ricovero e Cura a Carattere Scientifico (IRCCS) Humanitas Research Hospital, Milan, Italy; ^6^ Andalusian Center for Developmental Biology (CABD), Junta de Andalucía– Universidad Pablo de Olavide (UPO) – Consejo Superior de Investigaciones Cientificas (CSIC), Sevilla, Spain; ^7^ Center for Interdisciplinary Research on Medicines (CIRM) - Laboratory of Medicinal Chemistry, and Laboratory of Molecular Pharmacology, GIGA-Molecular Biology of Diseases, University of Liège, Liège, Belgium; ^8^ Laboratoire d’hormonologie Centre de Biologie et Pathologie Est (CBPE)-Groupement Hospitalier Est, Hospices civils de Lyon, Bron, France; ^9^ Department of Endocrinology, Centre Hospitalier Universitaire de Liège, University of Liège, Liège, Belgium; ^10^ Research Unit, Innovative Therapies for Endocrinopathies, Scientific Directorate, Bambino Gesù Children’s Hospital, Istituto di Ricovero e Cura a Carattere Scientifico (IRCCS), Rome, Italy

**Keywords:** gigantism, pituitary tumor, GPR101, topologically associating domain (TAD), somatostatin analog

## Abstract

X-linked acrogigantism (X-LAG) is a rare form of pituitary gigantism that is associated with growth hormone (GH) and prolactin-secreting pituitary adenomas/pituitary neuroendocrine tumors (PitNETs) that develop in infancy. It is caused by a duplication on chromosome Xq26.3 that leads to the misexpression of the gene *GPR101*, a constitutively active stimulator of pituitary GH and prolactin secretion. *GPR101* normally exists within its own topologically associating domain (TAD) and is insulated from surrounding regulatory elements. X-LAG is a TADopathy in which the duplication disrupts a conserved TAD border, leading to a neo-TAD in which ectopic enhancers drive *GPR101* over-expression, thus causing gigantism. Here we trace the full diagnostic and therapeutic pathway of a female patient with X-LAG from 4C-seq studies demonstrating the neo-TAD through medical and surgical interventions and detailed tumor histopathology. The complex nature of treating young children with X-LAG is illustrated, including the achievement of hormonal control using a combination of neurosurgery and adult doses of first-generation somatostatin analogs.

## 1 Introduction

Pituitary gigantism is a rare and severe form of acromegaly (1). It is caused by chronic excessive growth hormone (GH) and insulin-like growth factor 1 (IGF-1) secretion due to a pituitary adenoma and/or hyperplasia that begins before the growth plates have fused. Unlike in adult acromegaly, where germline genetic causes of pituitary adenomas are rarely encountered, nearly 50% of pituitary gigantism patients have an identifiable genetic cause (1–3). The most frequent of these are germline pathogenic variants (mutations) in the aryl hydrocarbon receptor interacting protein (*AIP*) gene, which account for around 30% of cases (2). Patients with pituitary gigantism due to *AIP* mutations are typically males that present during adolescence with large GH-secreting macroadenomas, which often co-secrete prolactin and may occur in familial isolated pituitary adenoma (FIPA) kindreds (4). The next most common genetic cause of pituitary gigantism is X-linked acrogigantism (X-LAG) (2). This very rare disease has a distinctive presentation beginning in the first 3 years of life (5). Most cases present with mixed GH and prolactin-positive macroadenomas, but individuals with hyperplasia alone have been described (5–7). In X-LAG, the young patients can have markedly increased height and weight at diagnosis compared with their age-peers (8). X-LAG is caused by duplications on chromosome Xq26.3 that include the gene *GPR101*, which encodes an orphan G protein-coupled receptor (GPCR) (5). In patients with X-LAG, there is a very marked over-expression of GPR101 in pituitary adenomatous/hyperplastic tissue (9). Usually, sporadic, X-LAG can occur due to constitutional or somatic mosaicism (in male patients) for the Xq26.3 duplication; three FIPA kindreds with X-LAG have also been reported (5, 10–13). As GPR101 is a constitutively active receptor that drives GH and prolactin secretion from somatotropes, this leads to hormonal hypersecretion and gigantism (5, 14, 15). Genomic duplications in X-LAG lead to disruption of the local chromatin structure around *GPR101*, which is normally insulated from nearby regulatory elements in its own topologically associating domain (TAD) (16). The duplication-induced reshuffling of genomic sequences and a TAD border brings the promoter of *GPR101* into contact with ectopic enhancers that form a neo-TAD and drive the over-expression of *GPR101* that typifies X-LAG (16).

While advances have been made in understanding the pathophysiology of X-LAG, the rarity of the disease means that there is incomplete information about longer-term responses to different management options. As X-LAG occurs in very young children, the management of pituitary adenoma is complicated in terms of surgical approach and choice of drug options and doses (5, 6, 8, 13, 17–20). Based on existing patients, many of whom were retrospectively diagnosed with X-LAG after many years of disease, GH and IGF-1 hypersecretion is challenging to control with standard approaches. New information on the long-term management of newly diagnosed X-LAG patients can help to clarify outstanding questions. In this study, we describe the presentation, diagnosis, and management of a young female patient with X-LAG from disease onset as a toddler until the age of nearly 10 years.

## 2 Methods

### 2.1 Pathology

Staining for anterior pituitary hormones (PRL, GH, and TSH), the transcription factor Pit-1, Ki67 (MIB1), somatostatin receptor 2A (SSTR2A), and low molecular weight cytokeratin CAM 5.2 was performed using a fully automated IHC stainer (BenchMark XT, Roche Ventana Medical System Inc.).

The antibodies for GH, prolactin, and TSH were rabbit polyclonal antibodies (Cell Marque) and were diluted at 1:100–1:500. The Pit-1 antibody was a rabbit POU1F1 antibody (Novus Bio) that was diluted 1:1,000–1:2,500. Ki67 (rabbit 30.9 antibody), and low molecular weight cytokeratin (mouse CAM 5.2) antibodies (Ventana) were supplied prediluted.

An immunoreactive score (IRS) was calculated for SSTR2A staining. This was generated by rating the staining intensity (no staining, 0; mild, 1; moderate, 2; strong, 3) and the percentage of cells showing a membranous or cytoplasmic expression (no positive cells, 0; 20% positive, 1; 40% positive, 2; 60% positive, 3; 80% positive, 4). The overall IRS was calculated as [percentage of positive cells] × [intensity of staining]. We considered the staining as being negative for IRS 0 and 1, weakly positive for IRS 2 and 3, moderately positive for IRS 4–8, and strongly positive for IRS >8.

### 2.2 4C-sequencing

The 4C library preparation was performed as previously described (21–23). The primer sequences, viewpoint fragment coordinates, and digestion strategies are as described in (16). For cell fixation experiments, approximately 1 to 2.5 × 10^6^ of leukocyte isolates from peripheral blood samples were used as input material for library preparation. These were trypsinized and filtered with a 40-μm cell strainer and pelleted by centrifugation at 500 × *g*. The cells were fixed using 5 mL of 2% formaldehyde in 10% FCS/PBS and incubated for 10 min at room temperature to cross-link the chromatin, after which the reaction was quenched, and the cells were again pelleted and washed on ice twice with 1× PBS before being snap-frozen for further preparation, cross-link reversal, DNA purification, restriction digestion, 4C library preparation, and analysis as outlined in (16).

## 3 Results

### 3.1 Clinical presentation and management

The patient was born at full term following an uncomplicated pregnancy. Her birth weight was 3.38 kg, and her length was 50 cm. Her parents had no history of overgrowth disorders (mid-parental height: 1.58 m); she has two older half-sisters who grew normally during childhood and adolescence. She had unremarkable development during the first year of life and met all of her physical and neuro-developmental milestones. When she was between 12 and 18 months of age, her mother noted that she was outgrowing children of the same age, and by her second birthday she was the tallest of her age-peer group. When the rapid growth began, the girl also had an increased appetite. She was reviewed regularly by her community pediatrician.

**Figure 1:**
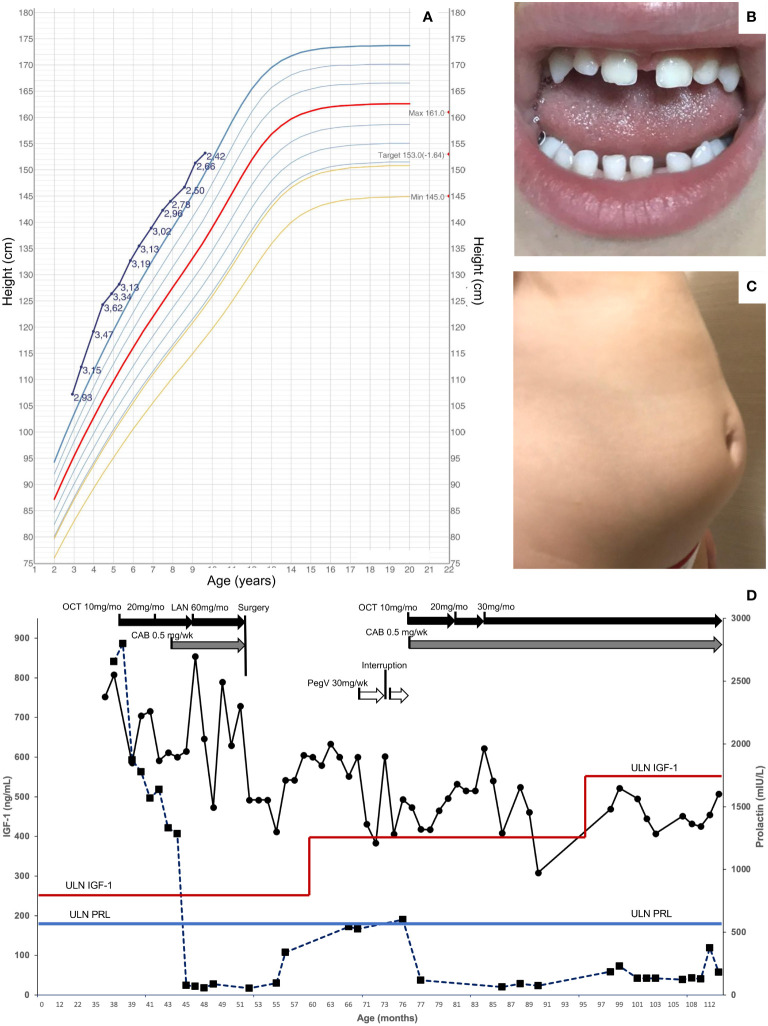
Clinical features in a patient with X-LAG. **(A)** Height chart from diagnosis to last follow-up in a female patient with X-LAG. Data derived from Italian reference dataset and standard deviation scores are shown alongside the individual growth curve (24). **(B)** Increased interdental spaces, and **(C)** abdominal lipohypertrophy associated with pegvisomant treatment. **(D)** Hormonal responses to treatment over time (IGF-1 on the left vertical axis, circles; prolactin on the right vertical axis, squares). The upper limit of normal for age and sex for IGF-1 is shown as a variable horizontal red line, while the upper limit for prolactin is shown as a horizontal blue line. CAB, cabergoline; i.m., intramuscular; LAN, lanreotide autogel; OCT, octreotide long-acting repeatable; pegV, pegvisomant; s.c., subcutaneous; ULN, upper limit of normal.

**Figure 2:**
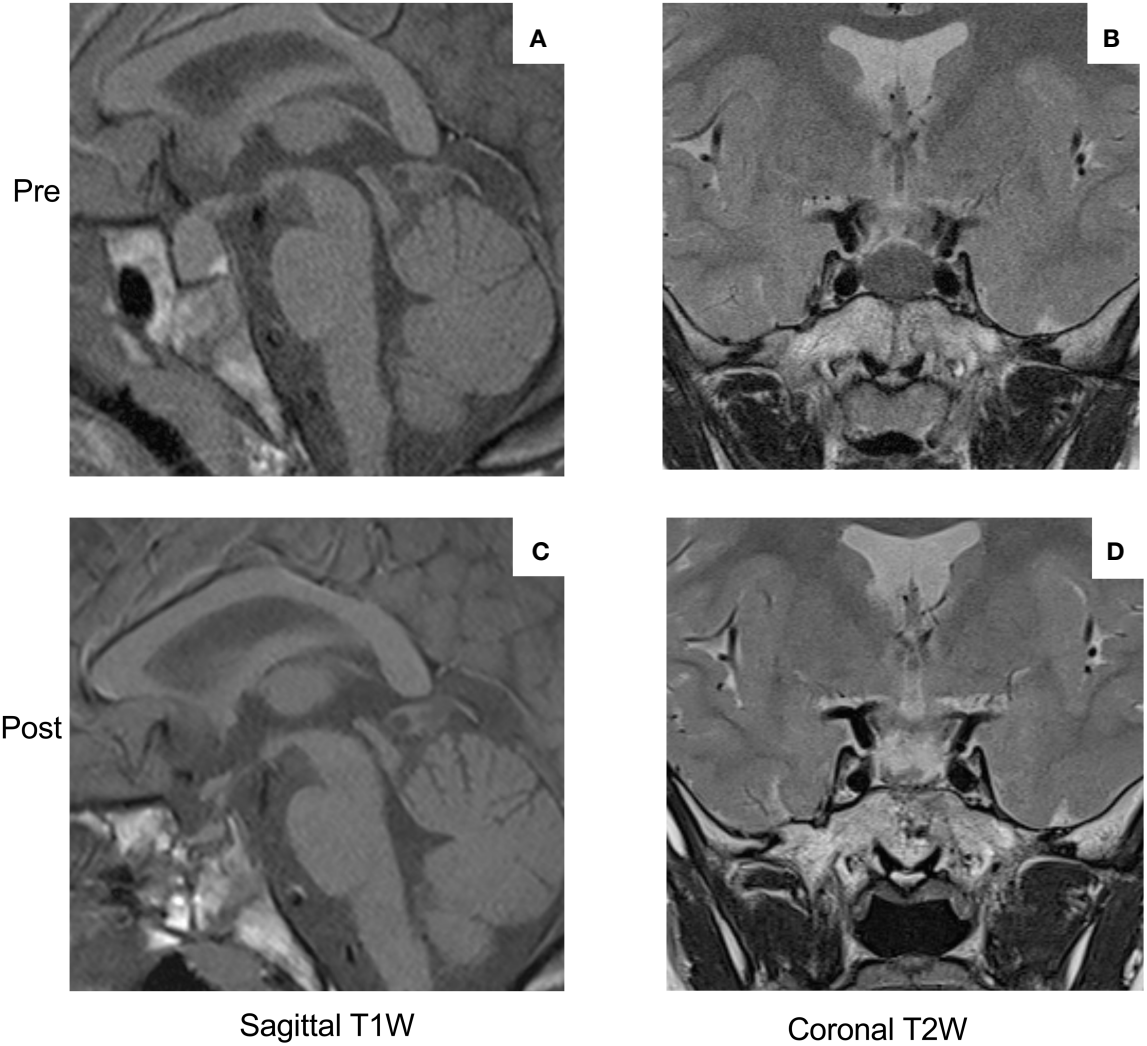
MRI images of the pituitary macroadenoma (17 × 12 mm) at diagnosis (sagittal T1-weighted image **(A)**, coronal T2-weighted image **(B)**). Note the hypo-intensity of the T2-weighted image as compared with the temporal lobe gray matter. The post-operative MRI images (**(C)** sagittal T1-weighted; **(D)** T2-weighted coronal)) show the effect of gross visual resection of the pituitary adenoma and a remaining tiny region of normal anterior pituitary tissue.

At 18 months of age, she was 82 cm in height and 11.9 kg in weight, which increased very rapidly to 92 cm and 14.05 kg by 21 months. Just before her third birthday, she was referred for growth assessment by a pediatric endocrinologist, at which time she was 107.2 cm (+2.93 SDS) in height and weighed 22.4 kg (+2.68 SDS; **Figure 1A**). She was hospitalized and, on examination, she had three small *café-au-lait* macules on her arm, leg, and buttock. The results of ophthalmological, abdominal ultrasound, and neurocognitive assessments were normal. A wrist X-ray showed a normal bone age for her chronological age. The hormonal testing showed very elevated levels of random GH (62 ng/mL), IGF-1 (752.1 ng/mL), and prolactin (2,656 mIU/L; 124.8 ng/mL) (**Figure 1D**). The bone scintigraphy result identified no areas that were suspicious for fibrous dysplasia. An MRI was performed, and the result revealed a pituitary mass measuring 17 × 12 mm that was hypointense on T2 series, suggestive of a pituitary adenoma/hyperplasia (**Figures 2A, B**). A tentative diagnosis of McCune–Albright syndrome complicated by a pituitary adenoma was made, and she was started on octreotide LAR 10 mg/month i.m. at the age of 38 months. The dose was titrated up to 20 mg/month i.m. after 4 months, but the GH and IGF-1 remained elevated, her rapid growth continued unabated, and the MRI findings were unchanged. Cabergoline was added (0.5 mg/week) and led to a rapid decrease in prolactin to within the normal range, but GH/IGF-1 were not affected (**Figure 1D**). Switching from octreotide LAR to lanreotide acetate had no impact on hormonal control or her increased growth. On examination, she developed increased interdental spaces (**Figure 1B**). The patient was referred for genetic studies, and an array CGH revealed a duplication at chromosome Xq26.3 (arr[hg19] Xq26.3(135638265_136294731) x3), which includes the *GPR101* locus (**Supplementary Figure 1**). A definitive diagnosis of X-LAG was made.

By the age of 4, her height had diverged further from normal values (+3.47 SDS). Due to the lack of efficacy of somatostatin analogs, the child was referred for neurosurgery. She underwent an endoscopic endonasal gross total resection of the adenoma. The patient had temporary arginine-vasopressin deficiency/diabetes insipidus and cortisol deficiency post-operatively, which resolved within 3–8 months. Her post-operative MRI results showed a small anterior pituitary remnant that appeared normal (**Figures 2C, D**). The GHRH levels were measured in the peri-operative period but were normal. The hormonal profile 5 months after surgery showed that both GH and IGF-1 remained elevated and that hyperprolactinemia had returned. Approval was sought for pegvisomant, which she began at a dose of 10 mg s.c. three times per week shortly before her sixth birthday. There was a drop in IGF-1, although interruption of drug supply meant that consistent control was not achieved. After 6 months, the patient developed lipohypertrophy of the abdomen and thighs (**Figure 1C**), a known adverse event associated with pegvisomant, and the treatment was stopped. Reintroduction of cabergoline led to the control of hyperprolactinemia, and she was restarted on octreotide LAR. Beginning at a dose of 10 mg/month, she was gradually up-titrated to the current level of 30 mg/month. During the last follow-up at the age of nine and a half years, she has not begun puberty and her IGF-1 is controlled (451.3 ng/mL; normal range: 49.0–549.0 ng/mL). As shown in **Figure 1A**, the growth curve for height is converging towards the 97th centile (currently +2.42 SDS), while her weight has been below the 97th centile since the age of 7.

### 3.2 Pathology

**Figure 3:**
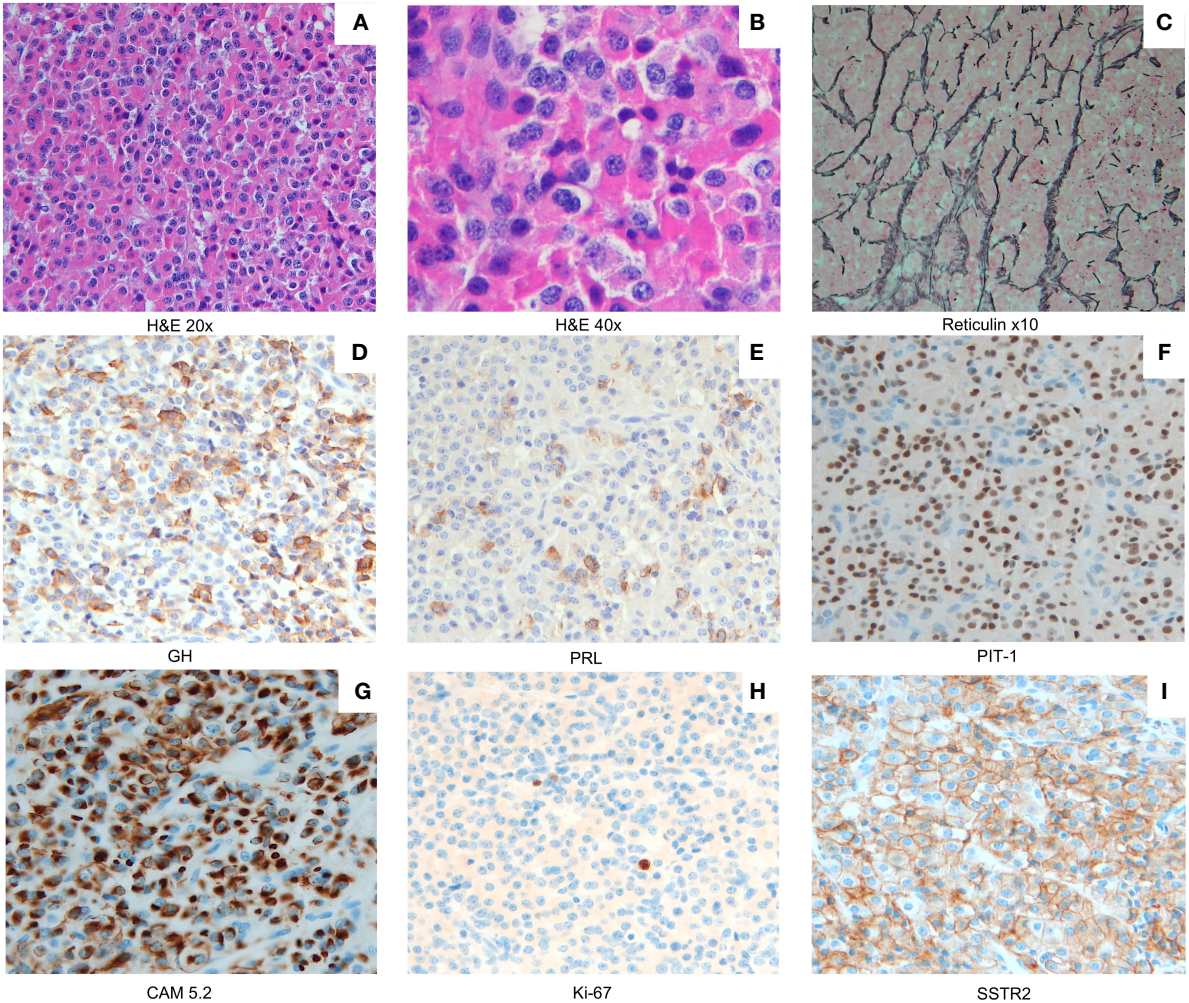
A mixed somatotroph–lactotroph PitNET/adenoma **(A)** composed of two distinct cell populations: somatotropes and lactotropes (×20 magnification). A combination of densely/sparsely granulated somatotropes mixed with sparsely granulated lactotropes was present (**(B)** ×40 magnification). Staining for reticulin fibers (**(C)** ×10 magnification) indicates the lobular and cordonal appearance of the X-LAG-related PitNEt/adenoma. Densely granulated (DG) somatotropes (GH staining **(D)**) represent the predominant component, while neoplastic lactotropes appear as smaller areas (PRL staining **(E)**). Less numerous and mostly interspersed among the acidophilic cells were slightly smaller chromophobic cells with eccentric nuclei. These sparsely granulated (SG) somatotrope cells showed a weak GH expression **(D)**. All tumor cellular components express Pit-1 **(F)**. A low molecular weight cytokeratin CAM 5.2 (CAM 5.2) immunostain revealed perinuclear expression in DG somatotropes **(G)** and dot-like expression of CAM 5.2 in fibrous bodies of SG somatotrophs **(G)**. The Ki67 labeling index was low (<2% **(H)**). Membranous and cytoplasmic SSTR2A staining was moderately positive (immunoreactive score, 4 out of 8 **(I)**). Magnification in **(D–I)**, ×20.

A histopathological examination of the surgically resected material revealed a mixed somatotrope–lactotrope cell pituitary adenoma/pituitary neuroendocrine tumor (PitNET) (**Figures 3A, B**) that was composed of distinct somatotrope and lactotrope populations. It was characterized by a predominantly sinusoidal and lobular pattern of growth, highlighted by a network of reticulin fibers (**Figure 3C**). The acidophilic cells were intermingled with separate areas of chromophobes. The acidophils displayed a large, highly eosinophilic cytoplasm. The nucleus was central and rounded in shape, with coarsely appearing chromatin. These cells displayed mild nuclear pleomorphism (**Figure 3B**). The mitotic activity was one mitosis per square millimeter. Neither necrosis nor hemorrhagic changes were observed. The acidophilic cells were GH-positive and were consistent with densely granulated (DG) somatotropes (**Figure 3D**). The chromophobic cells comprised a minority (<20%) of the tumoral tissue. The chromophobes were either positive for GH (**Figure 3D**) or prolactin (**Figure 3E**), and both were sparsely granulated. Pit-1 staining was positive in all tumoral cellular components (**Figure 3F**). The GH-positive chromophobes had fibrous bodies that were cytokeratin 5.2-positive (**Figure 3G**). The Ki67 labeling index was less than 2% (**Figure 3H**). The SSTR2a expression was variable, moderate (IRS score: 4/8), and both cytoplasmic and membranous (**Figure 3I**).

### 3.3 4C seq analysis

**Figure 4:**
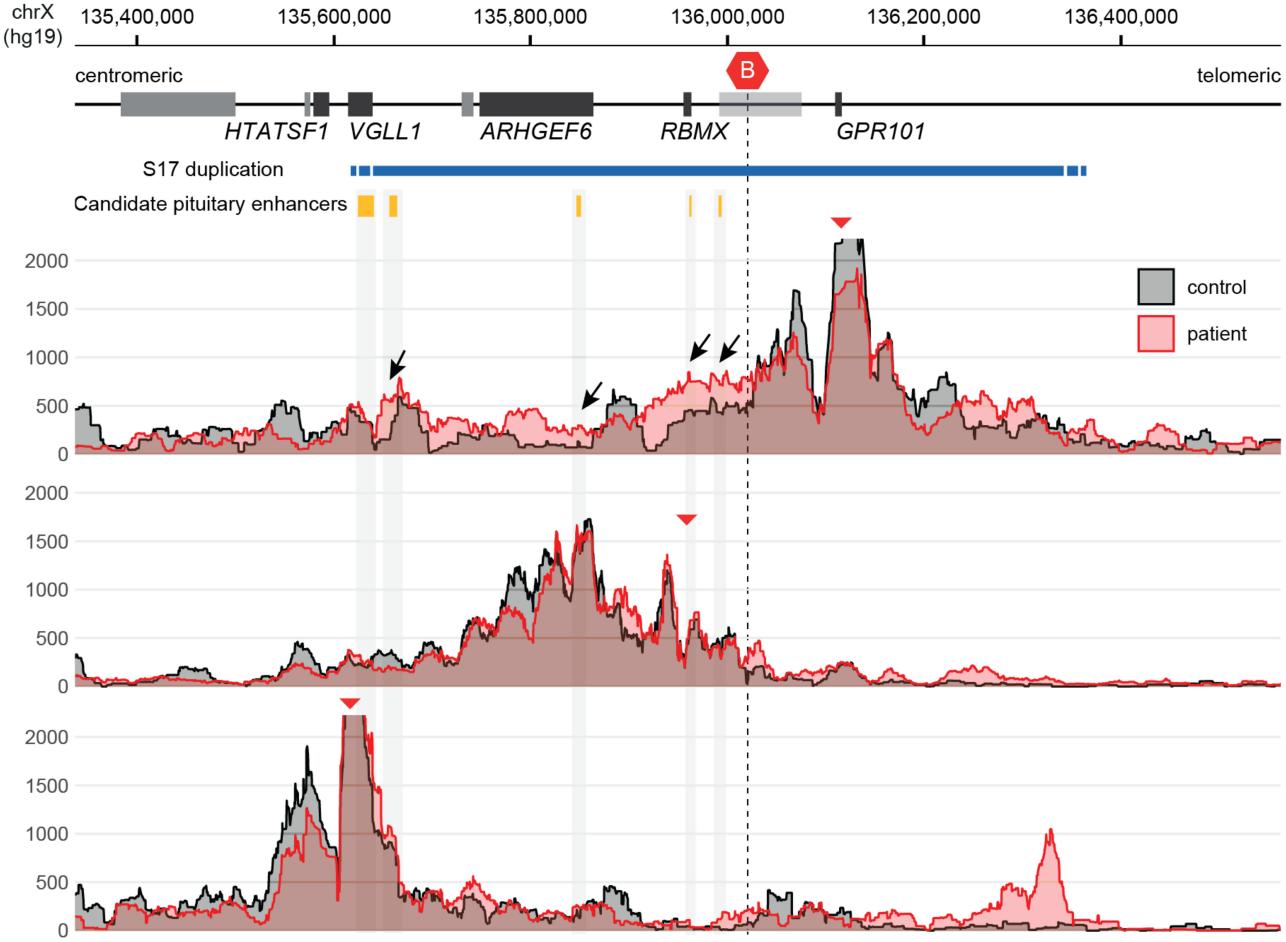
4C-seq showing ectopic chromatin interactions from *GPR101* due to the formation of a neo-TAD. Note the increase of *GPR101* interactions with potential pituitary enhancers (black arrows), while other duplicated genes show normal interaction patterns. The red triangles indicate the genomic position of viewpoints in the promoters of *GPR101*, *RBMX* and *VGLL1* (top to bottom). The conserved TAD border that is disrupted by duplications in X-LAG is highlighted by a red hexagon icon (“B”).

The 4C-seq analysis of chromatin derived from the patient’s peripheral blood leukocytes revealed an interaction pattern that was consistent with the loss of the invariant centromeric TAD border and the creation of a neo-TAD (16) (**Figure 4**).

## 4 Discussion

Somatotropinomas very rarely occur in early childhood but can have an important impact on the patient due to difficulties in treatment (1). The current study describes the complicated treatment journey that can occur in patients with the earliest-onset form of somatotropinomas, X-LAG. X-LAG is a very rare disease with fewer than 50 cases having been described in the decade since it was first identified as a distinct entity (2, 5, 6, 8, 10, 17–20, 25–29). The current patient illustrates well many of the characteristic features of X-LAG, namely, sporadic onset in a previously normally-growing female infant, with the first signs of overgrowth occurring around the first birthday. Over-growth usually becomes dramatic and drives patients onto the diagnostic pathway. By the time of the first MRI, X-LAG patients as young as 2 to 3 years old already have significant pituitary macroadenomas. Evidence indicates that tumorigenesis in X-LAG is already established perinatally. One familial case showed that excess GH and prolactin hypersecretion from an enlarged anterior pituitary (>10 mm in largest diameter) was clearly demonstrable by 3 weeks of age (10). Our patient had an established pituitary mass of 17 × 12 mm at the age of 4 years, which was later found to be a pituitary adenoma at surgery.

Hormonal hypersecretion in the current case was also typical of that seen in X-LAG in that it involved marked GH/IGF-1 and prolactin excess. While prolactin normalized rapidly with modest doses of cabergoline, the response of GH and IGF-1 to somatostatin analogs was poor during the pre-operative period. This was despite using adult-appropriate doses of octreotide and lanreotide in a child of only 3 to 4 years of age. Such resistance occurred despite a moderate-to-high expression of the SSTR2 receptor in the excised tumoral tissue, a factor that has been seen in previously reported X-LAG patients (8, 18). Other genetic forms of acrogigantism also show relative resistance to octreotide/lanreotide (30). Somatostatin analog resistance in patients with *AIP* mutations can have a number of explanations, both related to and independently of SSTR2 expression. A low AIP expression in mutated tissue could itself alter tumor responses to somatostatin analogs—for example, Bogner et al. showed that *AIP* mutations led to increased miR-34a, which, in turn, altered the inhibition of GH by octreotide independently of SSTR2 levels (31). X-LAG-related somatostatin resistance is not as yet fully explained. The circulating GH levels in these patients can be very high (5, 8, 18, 32). This may reflect a strong pro-secretory phenotype of the affected somatotropes potentially driven by protein kinase A and C-linked pathways in mice and in pituitary tumors from X-LAG patients (14). One potential mechanism for poor clinical responses to somatostatin analogs in patients is that treatment is not capable of overcoming this very high GH secretion driven by over-expressed, constitutively active GPR101 (14). In somatotropinoma patients who are resistant to first-generation somatostatin analogs, one of the main options is switching to pasireotide, which targets a wider number of somatostatin receptors (33–36). In the case of resistant acromegaly due to *AIP* pathogenic variants, it can be a valuable option, but not universally so, and appears to depend on tumoral somatostatin receptor expression (37, 38). In X-LAG, there is limited experience with pasireotide use and no clear evidence of improved hormonal control (26). Adjuvant radiotherapy has been used in the management of resistant hormonal hypersecretion in X-LAG, but it has the down-side of slow onset of effect, which limits its usefulness in controlling overgrowth promptly during childhood.

In X-LAG, there are two main therapeutic options that can bring about the effective control of GH hypersecretion and over-growth. The aggressive resection of the anterior pituitary or anterior hypophysectomy has been used to halt GH excess in some cases, particularly those with widespread hyperplasia (5). This comes at the obvious cost of panhypopituitarism, often accompanied by arginine–vasopressin deficiency, although these can be successfully replaced over the long term in X-LAG patients (5, 8). The other effective option is the use of the GH receptor antagonist, pegvisomant, which controls IGF-1 and growth in X-LAG (5, 8, 20). As illustrated by this case, X-LAG is remarkable in that tiny or even non-visualized post-operative tumoral remnants are capable of causing long-term GH-IGF-1 excess. The source of excess GH secretion in this and other such cases is likely to be GPR101 over-expressing cells contained within the residual normal-appearing pituitary tissue. In X-LAG and other forms of resistant pituitary gigantism, pegvisomant can be used to bring about rapid hormonal control and slow overgrowth, either alone or in combination with other medical therapies (2, 18, 20, 39–42). In the current case, pegvisomant could be used only briefly and despite an interruption of drug supplies, it successfully lowered the IGF-1 levels to close to normal. Lipohypertrophy at injection sites in the abdomen and thighs developed within 6 months of starting the pegvisomant treatment. This is a recognized side effect of pegvisomant for acromegaly, and as in other cases, the subcutaneous adipose tissue overgrowth regressed following drug withdrawal (43). As there was no overt tumor to target for re-operation or radiotherapy, this left very limited options. Cabergoline was reintroduced to lower prolactin, which was again effective. Preoperative treatment with relatively low doses of first-generation somatostatin analogs had been unsuccessful, so we hypothesized that tumor debulking (as in acromegaly) could permit hormonal control if we reintroduced octreotide LAR and dose-titrated upwards (44). The current study shows that it is possible to use post-operative, first-generation somatostatin analogs at adult doses to suppress GH excess from a non-visualized tumor remnant in X-LAG. Titration to 30 mg per month of octreotide LAR led to the control of circulating IGF-1, and the growth curve is now trending in the normal direction. As with other X-LAG cases, no tumor regrowth occurred following surgical resection (1, 5).

Pathological studies showed a Pit-1-positive mixed somatotrope–lactotrope adenoma or PitNET. Densely granulated, GH-positive acidophils predominated (80%) and were mixed with two sub-populations of sparsely granulated chromophobes—one was positive for prolactin and the other for GH. These findings are consistent with the pathological characteristics of X-LAG tumors (5, 8, 18). Adenomas are rarely accompanied by somatotrope hyperplasia, and a few cases have hyperplasia alone; no hyperplasia was seen in the tumor from the current patient (5, 8, 18). The tumor had a low Ki67 percentage, which is in line with most tumors from X-LAG patients, although a higher Ki67 level has been reported in one X-LAG family (5, 8, 10, 17, 18).

Initial genetic studies using aCGH confirmed that the patient had a 656.5-kb duplication on chromosome Xq26.3; neither of her parents carried this abnormality. The duplication included the last exon of *VGLL1* and the entire *CD40LG*, *ARHGEF6*, *RBMX*, and *GPR101* genes. We recently identified that duplications in this region cause X-LAG by interfering with a demarcated section of DNA called a topologically associating domain (TAD) (16). A TAD is a mega-base-scale genomic region within which interactions occur between genes and their regulators (enhancers and transcription factors) with higher likelihood than with factors outside of the TAD. This organization is facilitated by invariant boundaries that include binding sites for specific elements such as the transcriptional regulator CTCF, a key component of cohesin-mediated DNA looping and gene transcription. *GPR101* is normally contained within its own TAD and insulated from nearby genes and enhancers. However, duplications like those in X-LAG remove one of the invariant borders and create a neo-TAD that places the *GPR101* promoter under the control of ectopic pituitary enhancers located close to *VGLL1*, *ARGHEF6*, and *RBMX*. This drives the over-expression (>1,000-fold) of *GPR101* in somatotropes which is responsible for chronic GH, IGF-1, and prolactin hypersecretion that characterizes X-LAG. The study of leukocytes from the current patient demonstrated that her unique duplication led to a neo-TAD formation and ectopic enhancer interactions with the *GPR101* promoter (16).

In conclusion, this case illustrates the clinical presentation and complicated therapeutic journey in a sporadic female patient with infant-onset pituitary gigantism due to X-LAG. X-LAG is a very rare disorder due to *GPR101* over-expression that is driven by ectopic enhancers in a neo-TAD. This leads to a tightly choreographed presentation of very early-onset GH and prolactin-secreting adenomas that are resistant to treatment with preoperative first-generation somatostatin analogs. Disease persistence after gross total resection of the adenoma was eventually overcome with adult doses of octreotide, whereas pegvisomant had to be withdrawn due to lipohypertrophy. The combination of tumor debulking and adult doses of somatostatin analogs post-operatively represents a practical management option in pediatric patients with X-LAG.

## Data availability statement

The datasets presented in this study can be found in the GEO database under the accession code GSE249129.

## Ethics statement

The studies involving humans were approved by Liege University—CHU Liège Faculty Ethics Committee. The studies were conducted in accordance with the local legislation and institutional requirements. Written informed consent for participation in this study was provided by the participants’ legal guardians/next of kin. Written informed consent was obtained from the minor’s legal guardian/next of kin for the publication of any potentially identifiable images or data included in this article.

## Author contributions

MCar: Formal analysis, Investigation, Supervision, Writing – review & editing. DM: Investigation, Methodology, Writing – review & editing. SA: Data curation, Investigation, Methodology, Writing – original draft. GC: Data curation, Investigation, Writing – review & editing. GT: Data curation, Investigation, Writing – review & editing, Formal analysis. MF: Formal analysis, Investigation, Methodology, Writing – original draft. DA: Investigation, Writing – review & editing. JH: Investigation, Writing – review & editing. VR: Investigation, Writing – original draft. PP: Writing – review & editing, Supervision. AB: Investigation, Writing – review & editing. MCap: Investigation, Writing – review & editing. AD: Conceptualization, Data curation, Formal analysis, Investigation, Methodology, Supervision, Writing – original draft, Writing – review & editing.
