# Supplementary material for: Case report: Management of pediatric gigantism caused by the TADopathy, X-linked acrogigantism
Source: Front Endocrinol (Lausanne). 2024 Feb 28;15:1345363. doi: 10.3389/fendo.2024.1345363 (PMC10932951; doi:10.3389/fendo.2024.1345363)
Supplement: Supplementary file 1 [file Image_1.pdf]

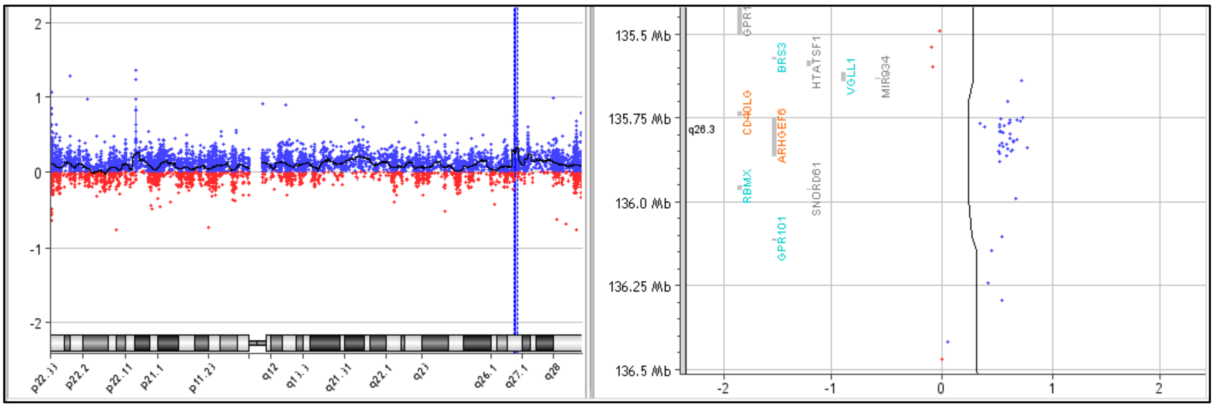

Supplemental Figure 1. Array comparative genomic hybridization (aCGH) showing chromosome Xq26.3 duplication involving *VGLL1* through *GPR101*.
